# Transactions of the Pennsylvania State Medical Society

**Published:** 1888-12

**Authors:** 


TRANSACTIONS OF THE MEDICAL SOCIETY OF THE State of Pennsylvania, 1888. Published by the Society.

This is a very elegant book of 351 pages filled with an unusual amount of very able and interesting articles. The publication is highly creditable to the society and the profession which it represents.

